# Uncovering Lipid Biomarkers Linked to Methylphenidate Efficacy in Treating Apathy in Alzheimer's Disease: Insights from the ADMET 2 trial

**DOI:** 10.1002/alz70856_105185

**Published:** 2026-01-07

**Authors:** Myuri Ruthirakuhan, Paul B. Rosenberg, Norman J Haughey, Jacobo Mintzer, Nathan Herrmann, Suzanne Craft, Alan J. Lerner, Allan I. Levey, Prasad R Padala, Anton P. Porsteinsson, Christopher H van Dyck, David Shade, Maya Mills, Krista L Lanctôt

**Affiliations:** ^1^ Hurvitz Brain Sciences Program, Sunnybrook Research Institute, Toronto, ON, Canada; ^2^ University of Toronto, Toronto, ON, Canada; ^3^ Johns Hopkins University School of Medicine, Baltimore, MD, USA; ^4^ Medical University of South Carolina, Charleston, SC, USA; ^5^ Wake Forest Alzheimer's Disease Research Center, Winston‐Salem, NC, USA; ^6^ Department of Neurology, Case Western Reserve University School of Medicine, Cleveland, OH, USA; ^7^ Goizueta Alzheimer's Disease Research Center, Emory University, Atlanta, GA, USA; ^8^ University of Arkansas for Medical Sciences, Little Rock, AR, USA; ^9^ University of Rochester School of Medicine and Dentistry, Rochester, NY, USA; ^10^ Yale School of Medicine, New Haven, CT, USA

## Abstract

**Background:**

Apathy is a prevalent neuropsychiatric symptom (NPS) in Alzheimer's disease (AD), linked to functional impairment and reduced quality of life. The Apathy in Dementia Methylphenidate Trial 2 (ADMET‐2) found modest efficacy of methylphenidate (MPH) for treating apathy, but treatment responses varied. This highlights the need for biomarkers to personalize treatments. Lipidomic profiling offers a promising approach by providing insights into the molecular basis of treatment response. Beyond their structural role in cell membranes, lipids serve as bioactive signaling molecules essential to neurotransmission, neuroinflammation, and synaptic plasticity—processes disrupted in NPS and AD. This study aimed to identify lipid species associated with MPH treatment response and explore lipid pathway disruptions in responders versus non‐responders.

**Method:**

Participants randomized to MPH in ADMET‐2 were analyzed. Responders were defined by a 4‐point improvement on the Neuropsychiatric Inventory Apathy subscale (NPI‐A). Baseline plasma samples underwent lipidomic profiling. Partial Least Squares Discriminant Analysis (PLS‐DA) was used to identify lipid species distinguishing responders from non‐responders, with model performance evaluated by area under the curve (AUC). Identified lipid species were analyzed in MetaboAnalyst for pathway enrichment.

**Result:**

A total of 45 participants were included, with 28 classified as responders. The PLS‐DA model achieved robust discrimination between responders and non‐responders, with an AUC of 0.81, indicating good predictive performance. Pathway analysis in MetaboAnalyst revealed disruption in lipid pathways related to ceramide, phosphospingolipid, and glycosphingolipid metabolism.

**Conclusion:**

This study demonstrates the utility of lipidomic profiling in identifying biomarkers of response to MPH in AD patients with apathy. The observed disruptions in ceramide, phosphosphingolipid, and glycosphingolipid metabolism suggest a role for sphingolipid signaling which could have effects on neurotransmission, neuroinflammation, and synaptic plasticity—key processes implicated in NPS and AD. The identified lipidomic species and pathways offer insights into the molecular mechanisms underlying treatment response and could inform future biomarker‐guided interventions.

